# Matrix metalloproteinase 9 facilitates Zika virus invasion of the testis by modulating the integrity of the blood-testis barrier

**DOI:** 10.1371/journal.ppat.1008509

**Published:** 2020-04-17

**Authors:** Lixia Hui, Yiwen Nie, Shihua Li, Moujian Guo, Wei Yang, Rui Huang, Junsen Chen, Yingxia Liu, Xuancheng Lu, Zhen Chen, Qingyu Yang, Ying Wu

**Affiliations:** 1 State Key Laboratory of Virology, School of Basic Medical Sciences, Wuhan University, Wuhan, China; 2 CAS Key Laboratory of Pathogenic Microbiology and Immunology, Institute of Microbiology, Chinese Academy of Sciences, Beijing, China; 3 Shenzhen Key Laboratory of Pathogen and Immunity, State Key Discipline of Infectious Disease, Second Hospital Affiliated to Southern University of Science and Technology, Shenzhen Third People’s Hospital, Shenzhen, China; 4 Laboratory Animal Center, Chinese Center for Disease Control and Prevention, Beijing, China; 5 Department of Tissue and Embryology, School of Basic Medical Sciences, Wuhan University, Wuhan, China; 6 Center for Translational Medicine, Wuhan Jinyintan Hospital, Wuhan, Hubei, China; 7 Hubei Province Key Laboratory of Allergy and Immunology, Wuhan, China; National Institute of Allergy and Infectious Diseases, UNITED STATES

## Abstract

Zika virus (ZIKV) is a unique flavivirus with high tropism to the testes. ZIKV can persist in human semen for months and can cause testicular damage in male mice. However, the mechanisms through which ZIKV enters the testes remain unclear. In this study, we revealed that matrix metalloproteinase 9 (MMP9) was upregulated by ZIKV infection in cell culture and in A129 mice. Furthermore, using an *in vitro* Sertoli cell barrier model and MMP9^-/-^ mice, we found that ZIKV infection directly affected the permeability of the blood-testis barrier (BTB), and knockout or inhibition of MMP9 reduced the effects of ZIKV on the Sertoli cell BTB, highlighting its role in ZIKV-induced disruption of the BTB. Interestingly, the protein levels of MMP9 were elevated by ZIKV nonstructural protein 1 (NS1) in primary mouse Sertoli cells (mSCs) and other cell lines. Moreover, the interaction between NS1 and MMP9 induced the K63-linked polyubiquitination of MMP9, which enhanced the stability of MMP9. The upregulated MMP9 level led to the degradation of essential proteins involved in the maintenance of the BTB, such as tight junction proteins (TJPs) and type Ⅳ collagens. Collectively, we concluded that ZIKV infection promoted the expression of MMP9 which was further stabilized by NS1 induced K63-linked polyubiquitination to affect the TJPs/ type Ⅳ collagen network, thereby disrupting the BTB and facilitating ZIKV entry into the testes.

## Author summary

Zika virus (ZIKV) is a flavivirus that shows high tropism to the testes and can persist in human semen for a long period. However, the entry mechanism of ZIKV into the testes has remained unclear. Here, we explored the mechanisms underlying matrix metalloproteinase 9 (MMP9)-modulated ZIKV infection in mice. We showed that MMP9 was upregulated by ZIKV infection both *in vivo* and *in vitro*. ZIKV infection affected the permeability of the blood-testis barrier (BTB) through MMP9 mediated degradation of TJPs and type Ⅳ collagens that are critically involved in the maintenance of the BTB. Additionally, the interaction between MMP9 and ZIKV NS1 induced the K63-linked polyubiquitination of MMP9, which enhanced the stability and function of MMP9.

Overall, our findings provided important insights into the mechanisms through which MMP9 disrupted the BTB and promoted ZIKV entry into the testes.

## Introduction

Zika virus (ZIKV), an emerging mosquito-borne virus, belongs to the genus *Flavivirus* within the family *Flaviviridae*. ZIKV was first identified from a sentinel rhesus monkey in Uganda in 1947 [1], and the first human infection was then described in this country in 1951 [2]. Since then, only sporadic cases of ZIKV were reported among Asian and African countries until the first epidemic occurred in 2007 in Micronesia, during which all reported cases were asymptomatic or manifested mild symptoms [3, 4]. The first large outbreak of ZIKV spread throughout the Pacific Ocean areas to American countries in 2013–2014, affecting approximately 30,000 people, and was shown to be associated with Guillain-Barré Syndrome, a severe neurological manifestation [2, 5, 6]. ZIKV infection was previously considered benign before recent retrospective studies and current outbreaks revealed strong causal associations with microcephaly in fetuses and newborns and other neurological disorders, attracting global attention. To date, ZIKV has spread to more than 84 countries worldwide [7], becoming a major public health concern. This concern has been further exacerbated by clinical evidence of potential transmission through sexual routes unique to ZIKV, in addition to mosquito bites described for other mosquito-borne flaviviruses in humans, such as dengue virus (DENV), Japanese encephalitis virus (JEV), and West Nile virus (WNV) [8].

Similar to other flaviviruses, ZIKV is a positive-sense, single-stranded RNA virus with a genome of 10.8 kb encoding a polyprotein that is processed into three structural proteins (capsid C, membrane protein M, and envelope protein E) and seven nonstructural proteins (NS1, NS2A, NS2B, NS3, NS4A, NS4B, and NS5) [9]. Upon systemic infection, ZIKV replication has been detected in most organs and several immune-privileged sites, such as the brain [10–12], placenta [13, 14], eye [15], testes, epididymis [16–21], and ovaries [22]. Unlike most other flaviviruses, ZIKV has high tropism to the testes and can cross the BTB, as demonstrated in murine models and human testes *ex vivo*. In these models, ZIKV infects and replicates in a variety of testicular cell types, including interstitial Leydig cells, Sertoli cells (SCs), and germ cells, and can cause testicular damage and reduction in motile sperms, resulting in infertility in male mice [16, 17, 19]. Several reports have revealed that SCs, which constitute the BTB, are highly susceptible to ZIKV infection, likely because they express high levels of AXL [23], a host molecule reported to be a possible attachment receptor or key factor promoting ZIKV infection [24]. Thus, SCs may act as a reservoir for long-term replication of virus in the testes, allowing ZIKV to continually infect germ cells and developing spermatocytes, even after peripheral clearance [25]. Recent studies have shown that ZIKV RNA can persist in human male semen up to 188 days after the onset of infection [23] illustrating its ability to pass through the BTB and disseminate into male reproductive tract [26]. Moreover, Govero et al. also found that the prolongation of ZIKV infection is accompanied by the loss of BTB integrity [16].

Mammalian testes are divided into the interstitial space and the seminiferous tubules [27]. The interstitial space contains testosterone-producing Leydig cells, immune cells, and blood vessels, whereas the seminiferous tubules consist of SCs, peritubular myoid cells, and spermatic cells at different stages of spermatogenesis [28]. During the normal process of spermatogenesis, earlier germ cells continually cross into the lumen of the seminiferous tubules by the restructuring of the BTB [29]. The BTB, also known as the SC barrier (SCB), is one of the tightest blood-tissue barriers [30]. The primary function of the BTB is to create an appropriate microenvironment to control germ cell development and maturation, provide an immunoprivileged environment, and shield germ cells from cytotoxic molecules [31]. In particular, the BTB is closest to the basement membrane, which is a type of pericellular matrix that is stabilized by a collagen type Ⅳ network [32]. The BTB is mainly composed of tight junctions (TJs) localized basally between adjacent SCs. Occludin, zonaoccludens (ZO)-1, and various claudins are the primary TJ proteins (TJPs) and are thought to be directly involved in this barrier mechanism [19]. Currently, the mechanisms underlying BTB invasion by ZIKV have not yet been fully elucidated.

Matrix metalloproteinases (MMPs) are calcium-dependent zinc-endopeptidases of the metzincin superfamily. MMPs play important roles in degrading basement membrane proteins and the extracellular matrix, thus facilitating tumor migration, cell infiltration, and angiogenesis [33]. MMP9 was previously reported to perturb various blood-tissue barriers. For example, MMP9 facilitates WNV entry into the brain by enhancing the blood-brain barrier permeability [34]. MMP9 also plays important roles in disrupting the blood-retinal barrier [35]. However, whether and how MMP9 is involved in ZIKV testis tropism remains unclear.

Accordingly, in this study, we evaluated the roles of MMP9 in ZIKV infection *in vivo* and *in vitro*. Our results provided insights into a novel mechanism through which ZIKV disrupted the integrity of the BTB to modulate entry into the testes, which might also account for the leakages of blood-brain barrier and other blood-tissue barriers by ZIKV infection.

## Results

### MMP9 expression and activity were induced by ZIKV infection *in vivo*

**Fig 1:**
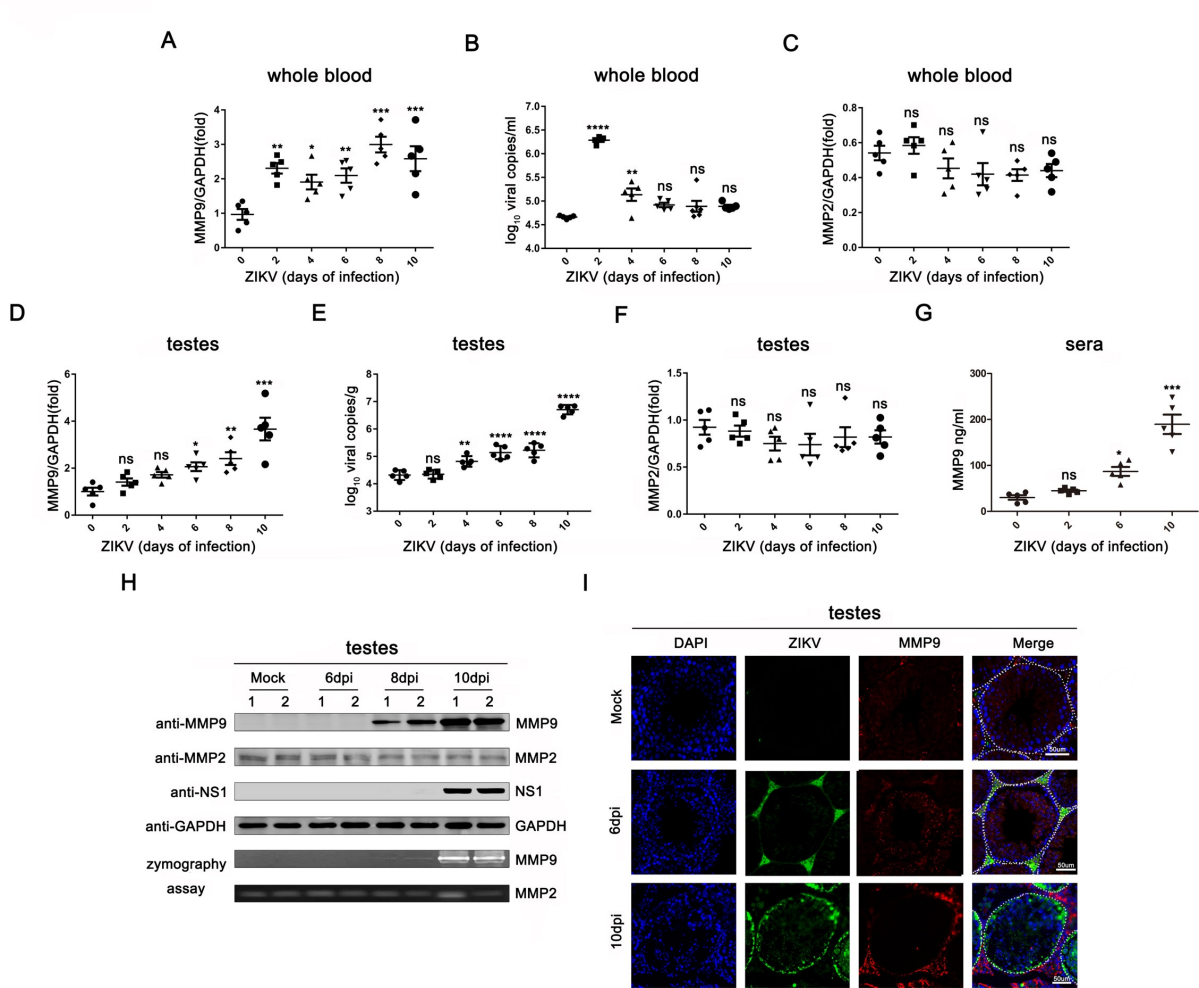
MMP9 was upregulated by ZIKV *in vivo*. A129 male mice (8 weeks old, n = 5) were infected with ZIKV (1×10^6^ PFU) for 0, 2, 4, 6, 8, or 10 days. **(A and C)** MMP9 mRNA expression levels **(A)** and MMP2 mRNA expression levels **(C)** in the whole blood were measured by qRT-PCR and normalized to the GAPDH mRNA level. **(B)** A probe-based assay was used to quantify viral RNA copy number by TaqMan qPCR amplification of ZIKV E gene. **(D and F)** MMP9 mRNA expression levels **(D)** and MMP2 mRNA expression levels **(F)** in the testes were examined by qRT-PCR. **(E)** A probe-based assay was used to quantify viral RNA copy number by TaqMan qPCR amplification of ZIKV E gene. **(G)** Protein levels of MMP9 in the sera were tested by ELISA. Data are expressed as means ± SEMS of three independent experiments. *P< 0.05; **P< 0.01; ***P< 0.001; ****P<0.0001. ns, not significant (one-way ANOVA). (**H**) Protein levels of MMP9, MMP2, NS1, GAPDH in the testes were quantified by western blotting (top), and proteinase activity was examined by gelatin zymography (bottom). Transparent bands indicate the presence of active MMP9 or MMP2. (**I**) Results of immunofluorescence staining. A129 mouse testis tissues were stained with DAPI to label nuclei (blue), an antibody against MMP9 (red), and ZIKV-positive signals (green) were detected by Z6 antibodies. Representatives of the tubules within the seminiferous epithelium are indicated with white dotted lines. Scale bar, 50μm.

Based on our previous quantitative proteomics analysis using testes of ZIKV-infected interferon α/β receptor deficient A129 mice, we found that among the most elevated proteins, MMP9 was upregulated by approximately 15-fold. To assess the expression of MMP9 during ZIKV infection *in vivo*, A129 mice were infected with ZIKV, and the expression of MMP9 was examined at the mRNA and protein levels at each time point. As shown in Fig 1A and 1B, when viremia in whole blood was evident in the circulation at day 2, MMP9 mRNA levels were also increased; however, when viremia markedly decreased on day 4, MMP9 mRNA levels steadily increased. Because MMP2 and MMP9 both cleave type Ⅳ collagens [36], we also tested MMP2 expression at the same time points. However, quantitative reverse transcription polymerase chain reaction (qRT-PCR) revealed that ZIKV infection did not affect MMP2 mRNA expression in whole blood (Fig 1C). Next, we examined the transcript levels of MMP9 in mouse testes. As shown in Fig 1D, MMP9 mRNA was upregulated from day 6 and peaked on day 10, showing a nearly 4-fold increase in expression. The testis viral load was highest on day 10 (Fig 1E). Similarly, MMP2 mRNA levels in testes were also unchanged (Fig 1F). Furthermore, we found that MMP9 protein levels were upregulated in sera following ZIKV infection (Fig 1G). Consistent with this, western blotting and gelatin zymography assays revealed that ZIKV infection, detected by NS1 expression, increased protein levels and gelatinase activity of MMP9 in the testes (Fig 1H), whereas MMP2 activity was not changed in infected testes, suggesting that MMP2 was constitutively expressed *in vivo* and was not altered by ZIKV infection. Similar results were found by immunofluorescence analysis. As shown in Fig 1I, mock (uninfected) mouse testes expressed low levels of MMP9 and no ZIKV fluorescence. At day 6, strong ZIKV positive signals were found in the interstitium, and much weaker but still detectable signals were found inside the seminiferous tubules, and the expression of MMP9 was slightly increased. Because ZIKV continuously replicated and spread in the testes, the virus signal was significantly higher at day 10, consistent with the increase in MMP9 staining. Moreover, during the course of infection, MMP9 was normally expressed in the seminiferous epithelium but then translocated to the interstitial spaces and the basement membrane, which was mainly composed of type IV collagens.

### MMP9 facilitated ZIKV entry into the testes by disrupting the BTB

**Fig 2:**
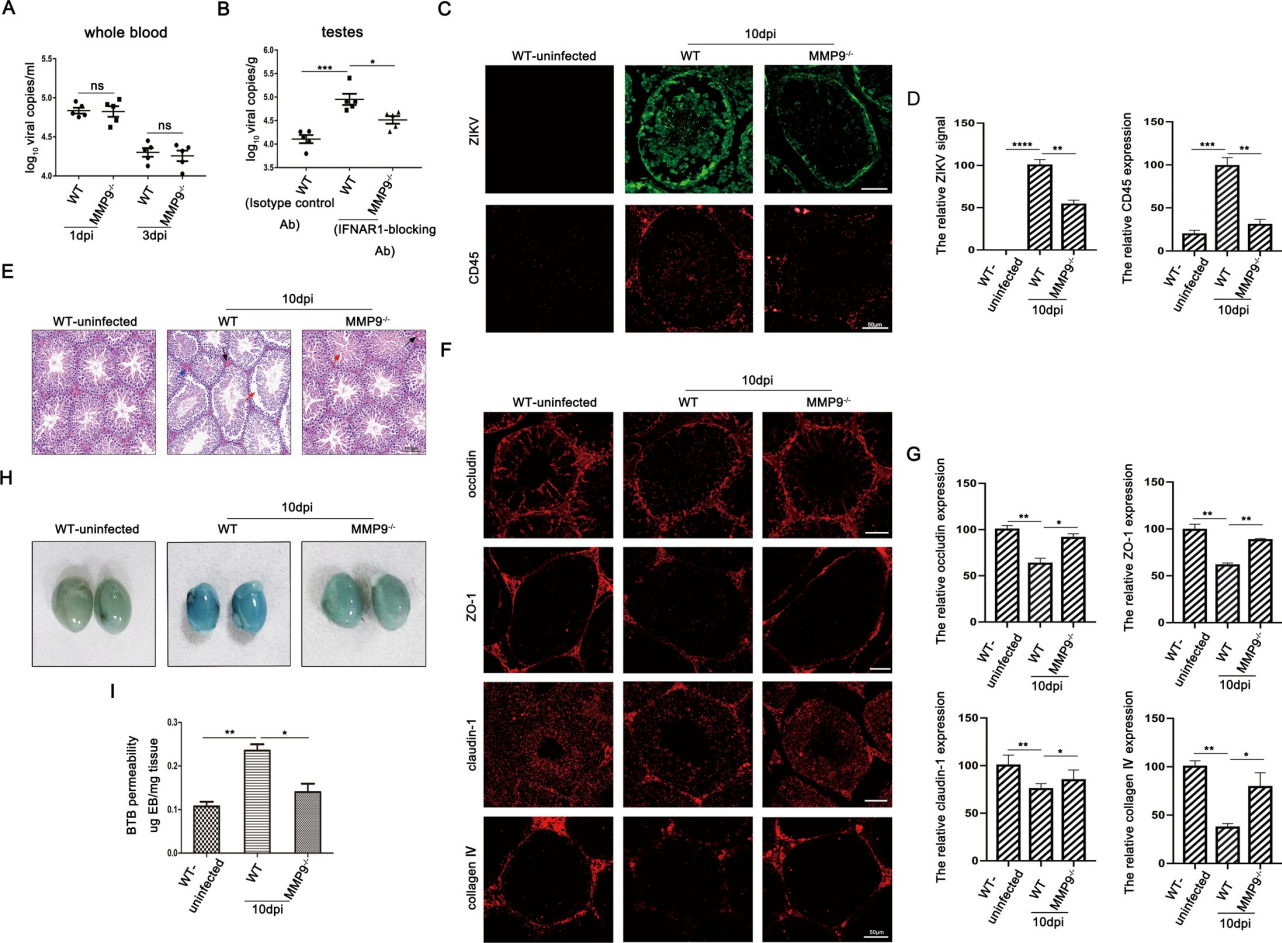
MMP9 compromised BTB integrity to facilitate ZIKV entry into the testes. C57BL/6 WT and MMP9^-/-^ male mice (6–7 weeks old) treated with Ifnar-blocking mouse monoclonal antibodies were infected intraperitoneally with ZIKV (1 × 10^7^ PFU). The C57BL/6 WT mice treated with isotype control antibodies were also infected intraperitoneally with ZIKV (1 × 10^7^ PFU) as a mock control. **(A)** RNA was extracted from the whole blood, and a probe-based assay was used to quantify viral RNA copy number by TaqMan qPCR amplification of ZIKV E gene at different time points. Data shown are means ± SEMs; ns, not significant, n = 5. (two-tailed Student’s t-tests). **(B)** RNA was extracted from the testes, and a probe-based assay was used to quantify viral RNA copy number by TaqMan qPCR amplification of ZIKV E gene at different time points. Data shown are means ± SEMs; *P< 0.05; **P< 0.01; ***P< 0.001, n = 5. (one-way ANOVA). **(C and D)** Results of immunofluorescence staining for ZIKV (green) and CD45 (red) in the testes and the quantifications were shown using Image J. Data shown are means ± SEMs; *P< 0.05; **P< 0.01; ***P< 0.001, ****P< 0.0001, n = 5 (one-way ANOVA), Scale bar, 50μm. (**E**) Histopathological changes in the testes of WT and MMP9^-/-^ mice on day 10 post infection. Disrupted seminiferous tubules with leukocyte infiltration (blue arrow), abnormally organized cells (red arrow), and intratesticular congestion (black arrow) were obviously observed in ZIKV-infected WT testes. Representative images from several independent experiments are shown, Scale bar, 100μm. (**F and G**) Results of immunofluorescence staining of occludin (red), ZO-1 (red), claudin-1 (red), and type Ⅳ collagen (red) and the quantifications for the percentage of these proteins were shown using Image J. Data shown are means ± SEMs; *P< 0.05; **P< 0.01, n = 5 (one-way ANOVA), Scale bar, 50μm. (**H**) Evans blue BTB permeability. On day 10 postinfection, WT and MMP9^-/-^ mice were injected with Evans blue and perfused 1h later. Uninfected WT mice were used as a control. Data are representatives of the results of three experiments (n = 8/group). (**I**) Quantification of Evans blue in the mouse testes. Evans blue was extracted from whole testes, and absorbance was measured, using uninfected-mouse testis extracts as a blank. *P< 0.05; **P< 0.01. ns, not significant (one-way ANOVA).

To identify the physiological relevance of elevated MMP9 in the pathogenesis of orchitis caused by ZIKV infection, we used Ifnar-blocked C57BL/6 wild type (WT) and C57BL/6 MMP9-knockout (MMP9^-/-^) mice as animal models. Firstly, the western blotting was used to verify that MMP9 is absent in the KO mice (S1A Fig). The viral loads both in whole blood and testes were monitored by qRT-PCR. As shown in Fig 2A, ZIKV infection in WT mice produced a short duration of viremia in the blood, with the highest level observed 1 day after infection and clearance observed at day 3; similar results were observed for MMP9^-/-^ mice. In contrast, testes of IFNAR1-blocking antibody treated-MMP9^-/-^ mice harbored a significantly lower viral load than those of treated WT mice at 10 days post infection (dpi) (Fig 2B). Additionally, as shown in Fig 2C and 2D, immunofluorescence analysis demonstrated that staining for ZIKV and CD45 in testis sections of infected MMP9^-/-^ mice was markedly weaker than that of infected WT mice at day 10; this was correlated with the higher viral loads and degree of inflammation in the testes of infected WT mice. Moreover, robust ZIKV signals were diffuse in the adluminal compartment as the BTB was perturbed in testis sections of infected WT mice. Subsequently, we examined histopathological changes in the testes of infected mice using hematoxylin and eosin (H&E) staining. As shown in Fig 2E, both WT and MMP9^-/-^ mice infected with ZIKV showed a certain degree of intratesticular varicoceles/ congestion and disordered spermatogenic cells compared with the uninfected WT group; however, the overall pathological characteristics of infected MMP9^-/-^mice were not as obvious as those of infected WT mice. Because disruption of the blood-tissue barrier during viral infection is mostly associated with the degradation or redistribution of TJPs [37], we assessed the staining of key TJPs, such as ZO-1, claudin-1, and occludin, by immunofluorescence analysis. As shown in Fig 2F and 2G, in the testes of ZIKV-infected WT mice, the fluorescence signals of TJPs were significantly weakened; this was not observed in the testes of MMP9^-/-^ mice infected with ZIKV. In addition, collagen Ⅳ was barely detected in infected WT mice, but was abundant in infected MMP9^-/-^ mice, suggesting that ZIKV infection resulted in MMP9-mediated loss of collagen IV. Additionally, we also performed western blotting to quantify these protein levels (S1B Fig). Finally, leakage of Evans blue into the testes of perfused infected MMP9^-/-^ mice was much lower than in infected WT mice but comparable to that in uninfected WT mice (Fig 2H and 2I). These results collectively suggested that MMP9 facilitated ZIKV entry into the testis by disrupting the BTB.

### ZIKV infection increased the permeability of an *in vitro* SCB model

**Fig 3:**
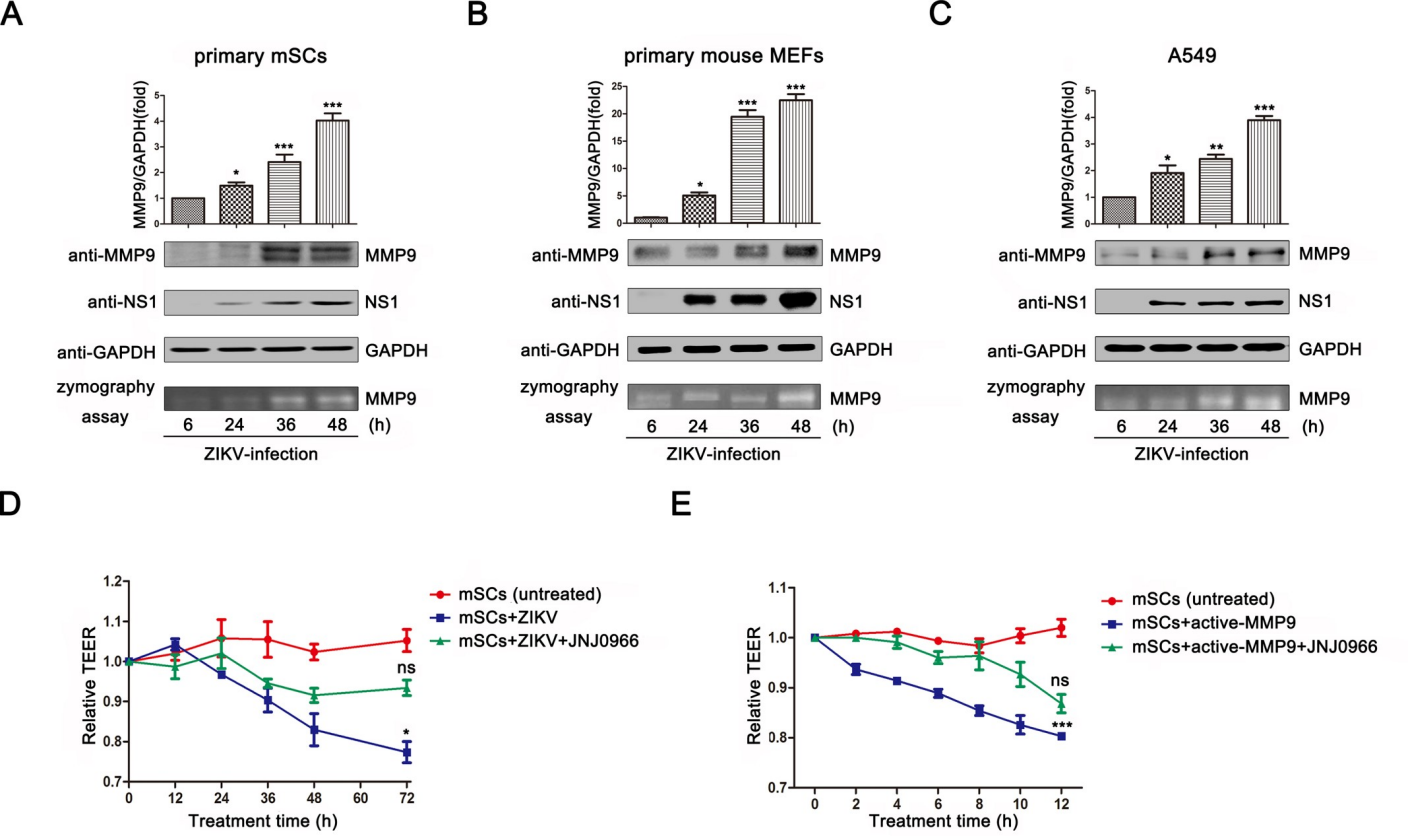
ZIKV infection induced the expression and activity of MMP9 and altered barrier integrity. **(A**–**C)** Primary mSCs (**A**), primary MEFs (**B**), and A549 cells (**C**) were infected with ZIKV at a MOI of 1. MMP9 mRNA levels were measured by quantitative RT-PCR (top), MMP9 protein levels were examined by western blotting (middle), and MMP9 proteinase activity was determined by gelatin zymography assays (bottom). Data are expressed as means ± SEMs of three separate experiments. *P< 0.05; **P< 0.01; ***P< 0.001. ns, not significant (one-way ANOVA). **(D)** Primary mSCs were cultured on Transwell semipermeable membranes (0.4 μm pore size). At day 3, Sertoli cells with an established functional tight junction barrier were infected with ZIKV (MOI = 5) and then treated with or without a specific inhibitor of MMP9 (JNJ0966; 1μM). The untreated cells were used as a mock control. The integrity of the SCB model was determined by measuring TEER at each time point. TEER values were expressed in Ohms (Ω). **(E)** Primary mSCs were cultured on Transwell semipermeable membranes (0.4 μm pore size), activated MMP9 (50ng/mL) were added and then treated with or without JNJ0966 (1μM). The integrity of the SCB model was determined by measuring TEER at each time point. The data are expressed as means ± SEMs of three independent experiments. *P< 0.05; **P< 0.01; ***P< 0.001. ns, not significant (one-way ANOVA).

MMP9 can perturb various blood-tissue barriers [34, 38]. Therefore, we further investigated the effects of ZIKV on the permeability of the SCB *in vitro*. Here, we initially measured whether ZIKV infection could induce the expression and activity of MMP9 *in vitro*. The expression and activity of MMP9 in primary mSCs (Fig 3A), primary mouse embryonic fibroblasts (MEFs; Fig 3B), and adenocarcinomic human alveolar basal epithelial cells (A549) (Fig 3C) were measured after ZIKV infection. We observed increases in MMP9 mRNA levels by qRT-PCR. Further analysis by western blotting showed significant upregulation in MMP9 protein levels with the increase of NS1. Moreover, gelatin zymography assays showed that MMP9 enzyme activity was elevated in all three cell types as the concentration of MMP9 increased, confirming that the induced MMP9 had metalloproteinase activity. We also examined the expression of MMP9 in human embryonic kidney cells (HEK293T) after ZIKV infection at different multiplicity of infection (MOI) (S2A Fig). Taken together, these findings demonstrated that MMP9 was upregulated both in time-dependent and ZIKV-dose dependent manners by ZIKV infection.

According to previous studies, transepithelial electrical resistance (TEER) is widely implicated as a marker for the integrity of the BTB [38], and decreased TEER in cells indicates the hyperpermeability of BTB. Thus, because SCs are central to the maintenance of BTB integrity, we next utilized primary mSCs as an *in vitro* SCB model to evaluate TEER. As shown in Fig 3D, mSCs infected with ZIKV (MOI = 5) showed a significant loss in TEER compared with the untreated cells at 72h postinfection, indicating that ZIKV could increase the permeability of primary mSCs. This decline in TEER was not due to a reduced cell viability because we observed that primary mSCs can support ZIKV infection without causing any cytopathic effect or cell death (S2C Fig). However, this decline was attenuated in the presence of an MMP9-specific inhibitor. Moreover, we also added 50 ng/mL activated MMP9 into the upper chambers of cell cultures, as shown in Fig 3E, cells treated with the activated MMP9, compared with untreated mSCs, showed a dramatic reduction in TEER values with no negative effect on cell viability (S2D Fig), and this effect was blocked by treatment with an MMP9-specific inhibitor (JNJ0966) (Fig 3E). A gelatin zymography assay was performed to demonstrate that the inhibitor blocks the enzymic activity of MMP9 (S2B Fig). Collectively, these results suggested that MMP9, induced by ZIKV infection, caused SCB hyperpermeability.

### MMP9 was upregulated by ZIKV NS1

**Fig 4:**
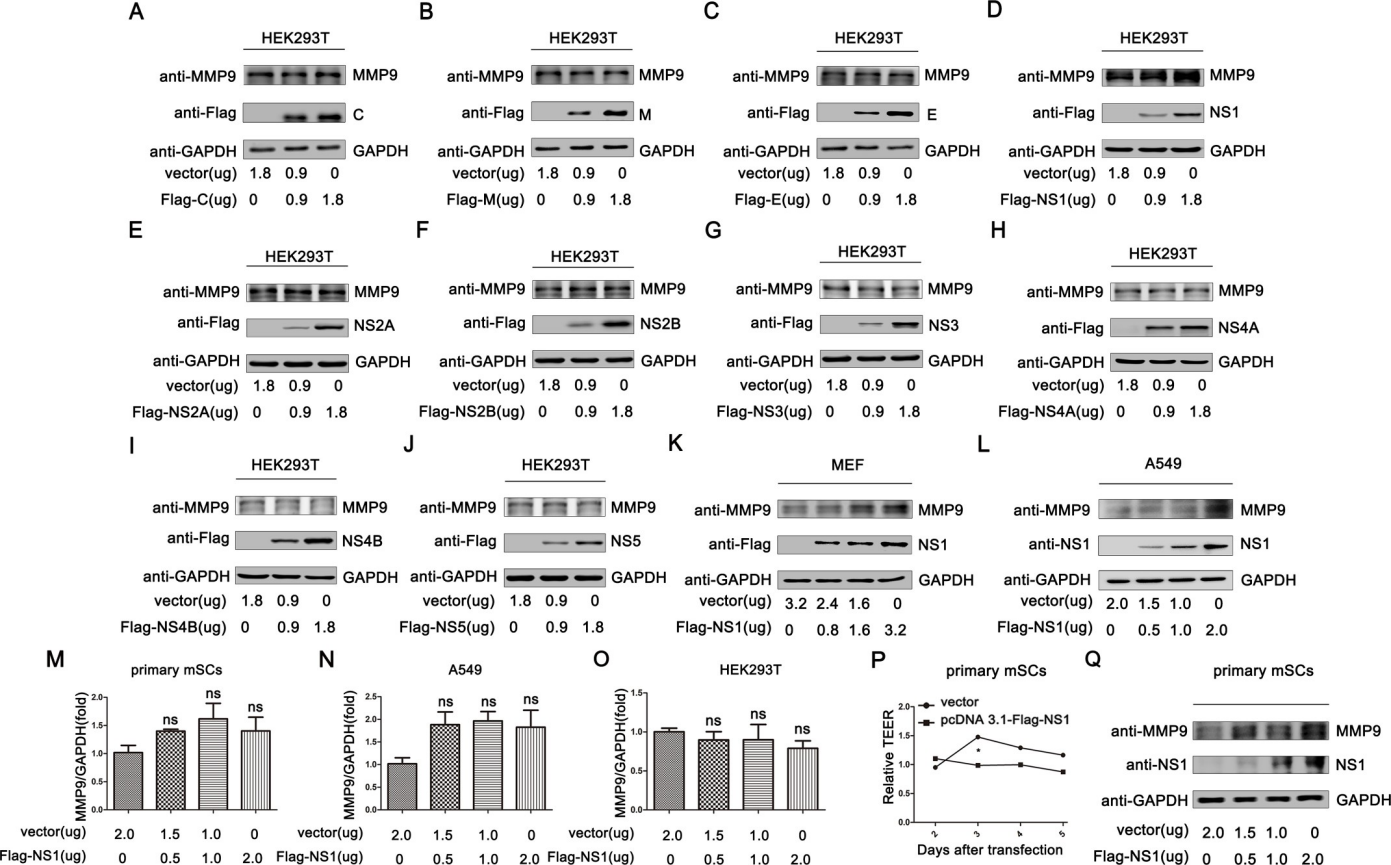
MMP9 was upregulated by ZIKV NS1 on protein level. **(A**–**J)** HEK293T cells were transfected with pcDNA3.1(+)-Flag-C, -M, -E, -NS1, -NS2A, -NS2B, -NS3, -NS4A, -NS4B, or -NS5 using a concentration gradient for 48h. Total MMP9, C, M, E, NS1, NS2A, NS2B, NS3, NS4A, NS4B, NS5, and GAPDH levels in whole cell lysates (WCLs) were determined by western blotting with the indicated antibodies. MEFs (**K)** and A549 cells **(L)** were transfected with different concentrations of pcDNA3.1(+)-Flag-NS1. After 48h post tranfection total MMP9, NS1, and GAPDH proteins expressed in WCLs were detected by western blotting with the indicated antibodies. **(M**–**O)** Different concentrations of pcDNA3.1(+)-Flag-NS1 were transfected into primary mSCs (**M**), A549 cells (**N**), and HEK293T cells (**O**). After 48h post tranfection, MMP9 mRNA levels were measured by qRT-PCR. Data are expressed as means ± SEMs of three independent experiments. ns, not significant (one-way ANOVA). (**P**) Primary mSCs were transfected with empty vector or pcDNA3.1(+)-Flag-NS1, and resistance values were examined from day 2 after transfection. Data are expressed as means ± SEMs of three independent experiments. *P< 0.05. ns, not significant (two-way ANOVA). (**Q**) Primary mSCs were transfected with empty vector or pcDNA3.1(+)-Flag-NS1, and the expression of NS1 and MMP9 was measured by western blotting.

To investigate the mechanisms through which ZIKV facilitated MMP9 expression and then caused SCB hyperpermeability, we initially determined whether ZIKV proteins were related to MMP9 upregulation. Therefore, three plasmids encoding structural proteins of ZIKV (C, M, and E) and seven plasmids encoding nonstructural proteins of ZIKV (NS1, NS2A, NS2B, NS3, NS4A, NS4B, and NS5) were transfected into HEK293T separately in a concentration-gradient analysis. Interestingly, the endogenous expression of MMP9 was significantly upregulated by NS1 in a concentration-dependent manner. However, the other proteins failed to facilitate the expression of MMP9 (Fig 4A–4J). Next, we further confirmed the results in MEFs (Fig 4K) and A549 cells (Fig 4L). We found that MMP9 was also elevated as the concentration of NS1 increased. At the same time, we also tested whether the addition of recombinant NS1 protein can increase the expression of MMP9 protein. As shown in S3A Fig, the expression of MMP9 did not change when recombinant NS1 protein was added to the cell culture supernatant. In addition, we used primary mSCs (Fig 4M), A549 cells (Fig 4N), and HEK293T cells (Fig 4O) to detect whether NS1 could upregulate the mRNA expression of MMP9. The results showed that NS1 had no effect on the transcription of MMP9. Taken together, MMP9 was upregulated by the intracellular overexpression of ZIKV NS1 on protein level.

In order to determine the effects of NS1 on the permeability of SCB by upregulation of MMP9 expression, we transfected primary mSCs with an NS1 plasmid or empty vector to detect TEER values. The results showed that the resistance values in cells transfected with NS1 failed to increase, whereas those in cells transfected with empty vector gradually increased, further forming tight junctions. These findings suggested that NS1 had negative effects on the formation of tight junctions by upregulating MMP9 (Fig 4P). To confirm the transfection efficiency, we detected the expression levels of NS1 and MMP9 by western blotting (Fig 4Q). Collectively, our findings suggested that NS1 protein, rather than the other proteins, led to upregulation of MMP9 and caused leakage of SCB.

### NS1 bound to MMP9 and facilitated its stability by inducing K63-linked polyubiquitination of MMP9

**Fig 5:**
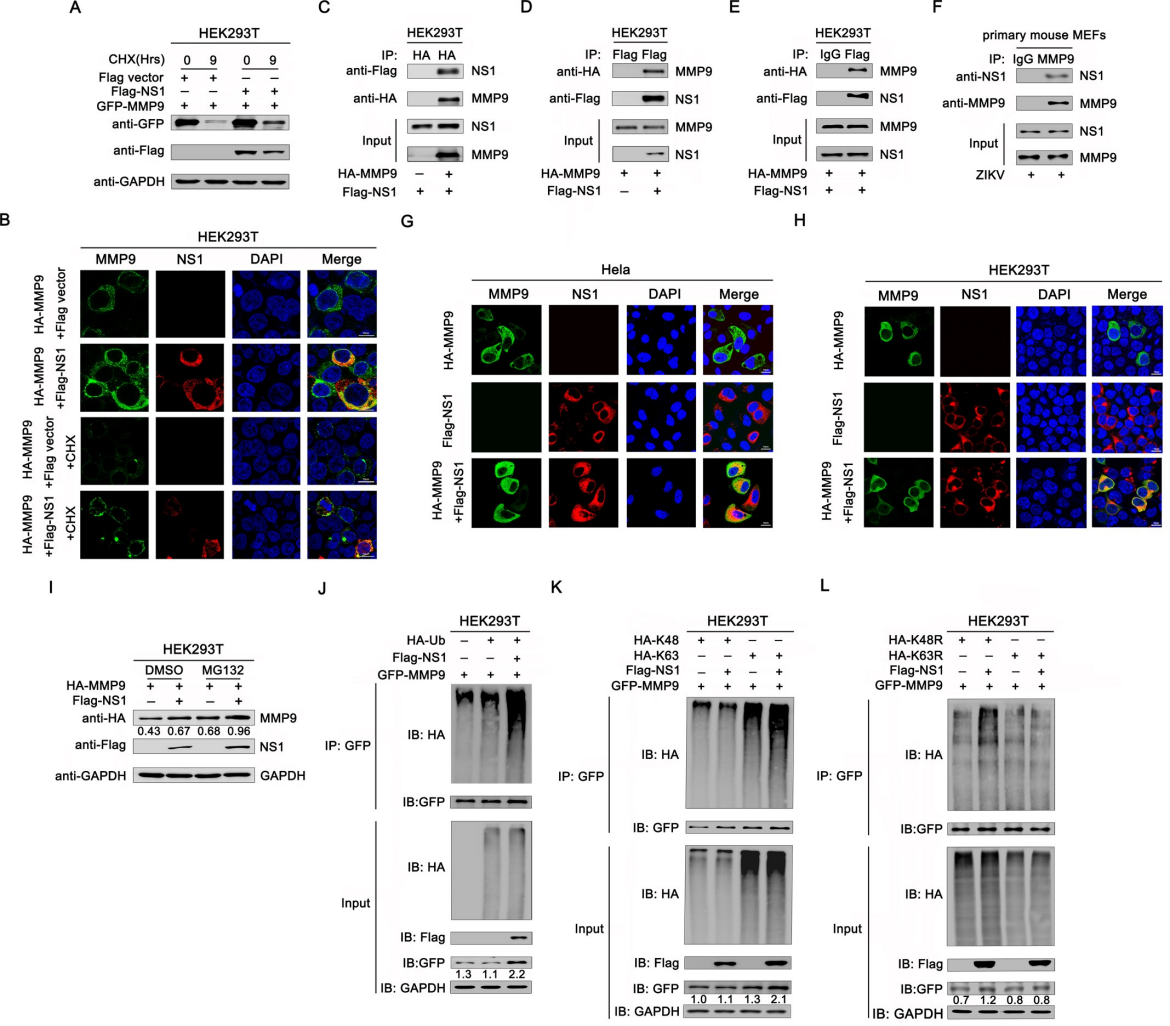
NS1 bound to MMP9 and facilitated K63-linked polyubiquitination of MMP9. (**A)** HEK293T cells were cotransfected with empty vector, plasmid pcDNA3.1(+)-Flag-NS1 expressing Flag-tagged NS1 and plasmid pCAGGS-HA-MMP9 expressing HA-tagged MMP9. 48h later, cells were treated with 40μg/mL CHX for 0, 9h, and then cells were lysed, and subjected to western blot with indicated antibodies. **(B)** HEK293T cells were cotransfected with empty vector/ Flag-NS1 and HA-MMP9 for 30h. Cells were treated with 40μg/mL CHX for 0, 9h, then immunostained with anti-Flag (red) and anti-HA (green) antibodies, and nuclei were stained with DAPI (blue) and analyzed by confocal microscopy. Scale bar, 10μm. **(C-D)** HEK293T cells were cotransfected with empty vector, Flag-NS1 and HA-MMP9, cells lysates were immunoprecipitated with anti-HA **(C)** or anti-Flag **(D)** antibodies. The immunoprecipitates and whole-cell lysates (WCLs) were analyzed by western blotting with anti-Flag and anti-HA antibodies. **(E)** HEK293T cells were cotransfected with plasmid Flag-NS1 and HA-MMP9. Cells were lysed, and cell lysates were immunoprecipitated with anti-Flag and anti-mouse immunoglobulin G (IgG) antibodies. The immunoprecipitates and whole-cell lysates (WCLs) were analyzed by western blotting with anti-Flag and anti-HA antibodies. (**F**) Primary MEFs were infected with ZIKV at an MOI of 1 for 30h, and cell lysates were immunoprecipitated with anti-MMP9 and anti-goat IgG antibodies. The immunoprecipitates and WCLs were analyzed by western blotting with anti-MMP9 and anti-NS1 antibodies. HeLa (**G**) and HEK293T cells (**H**) were cotransfected with Flag-NS1 and HA-MMP9 for 24h. Cells were immunostained with anti-Flag (red) and anti-HA (green) antibodies, and nuclei were stained with DAPI (blue) and analyzed by confocal microscopy. Scale bar, 10μm. **(I)** Immunoblot analysis of extracts of HEK293T cells transfected with plasmid for HA-MMP9 and Flag-NS1 and treated with dimethylsulfoxide (DMSO) or MG132. (**J**) HEK293T cells were cotransfected with pGFP-MMP9, pHA-Ub, and pFlag-NS1. Cell lysates were immunoprecipitated with anti-GFP antibodies and immunoblotted with anti-HA antibodies. (**K)** HEK293T cells were cotransfected with pGFP-MMP9, pHA-K63, and pHA-K48, together with pFlag-NS1. Cell lysates were immunoprecipitated with anti-GFP and immunoblotted with anti-HA antibodies. (**L)** HEK293T cells were cotransfected with pGFP-MMP9 and pHA-K63R or pHA-K48R, together with pFlag-NS1. Cell lysates were immunoprecipitated with anti-GFP antibodies and immunoblotted with anti-HA antibodies.

Since we observed an increased protein level of MMP9 after NS1 overexpression as shown in Fig 4, we performed protein stability assays by expressing MMP9 with or without NS1 in HEK293T cells to demonstrate whether NS1 can regulate MMP9 at the protein level. Cells were treated with cycloheximide (CHX, a translation inhibitor) for up to 9h. The results showed that the protein level of MMP9 was decreased dramatically in the absence of NS1 (Fig 5A). Additionally, we used confocal microscopy to examine the stabilizing effect of NS1 on MMP9. As shown in Fig 5B, the staining of MMP9 was markedly stronger in the presence of NS1, which is consistent with the conclusions above. When CHX was added, the fluorescence signal of the protein was greatly weakened due to the inhibition of translational synthesis. However, it was still obvious that the fluorescence signal of MMP9 was stronger in the presence of NS1 than in the absence of NS1. Thus, NS1 stabilized MMP9 protein by delaying its degradation. We further investigated whether NS1 can interact with MMP9. Co-immunoprecipitation (Co-IP) results showed that NS1 was co-immunoprecipitated with MMP9, suggesting an interaction between NS1 and MMP9 (Fig 5C–5E). Additionally, primary MEFs were infected with ZIKV (MOI = 1), and Co-IP revealed that endogenous MMP9 was also precipitated with NS1 (Fig 5F), consistent with the results of cotransfection with NS1 and MMP9 expression plasmids. In addition, considering that both MMP9 and NS1 are secreted proteins, we have also performed Co-IP assay to measure the interaction between MMP9 and NS1 proteins in the cell-culture supernatant. The results demonstrate an interaction between these secreted proteins (S4 Fig). Moreover, confocal microscopy was used to detect the cell distribution of NS1 and MMP9. As shown in Fig 5G and 5H, both NS1 and MMP9 were predominantly located in the cytoplasm in HeLa and HEK293T cells cotransfected with NS1 and MMP9 expression plasmids. The colocalization between NS1 and MMP9 suggested a potential association between these two proteins. Further, as shown in Fig 5I, the protein level of MMP9 was up-regulated when the cells were treated with the proteasome inhibitor MG132 compared with the non-treated (Lane 3 vs. 1), which indicated that the degradation of MMP9 is via the proteasome pathway. Meanwhile, the expression of MMP9 in cells cotransfected with NS1 was similar to that of the cells treated with MG132 (Lane 2 vs. 3), and the protein level of MMP9 in cells also expressing NS1 was obviously higher than that of the cells with no NS1 expressing when the proteasome activity was inhibited by MG132 (Lane 4 vs. 3). Collectively, NS1 promotes MMP9 stability by preventing proteasome degradation of MMP9.

Ubiquitination is a type of post-translational modification that plays critical roles in the regulation of various physiological responses in host cells. It was reported that K63-polyubiquitin can prevent proteins from binding proteasome for degrading [39]. Here, to examine whether NS1 had a role in MMP9 ubiquitination, MMP9 plasmids were co-expressed with hemagglutinin (HA)-ubiquitin (Ub) and Flag-NS1 or an empty vector. MMP9 polyubiquitination was catalyzed by HA-Ub (Fig 5J, lane 2), and upregulated by NS1 (Fig 5J, lane 3). Additionally, MMP9 polyubiquitination was strongly catalyzed by HA-K63 (HA-tagged Ub mutant retaining a single lysine at K63; Fig 5K, lane3), and this K63-linked polyubiquitination was further promoted by NS1 (Fig 5K, lane 4); however, MMP9 polyubiquitination was weakly catalyzed by HA-K48 (HA-tagged Ub mutant retaining a single lysine at K48; Fig 5K, lane 1), and MMP9 K48-linked polyubiquitination was not affected by NS1 (Fig 5K, lane 2). Moreover, MMP9 polyubiquitination was weakly catalyzed by K63R (Ub mutant with lysine 63 mutated to arginine; Fig 5L, lane 3), which was not affected by NS1 (Fig 5L, lane 4). In contrast, MMP9 polyubiquitination was catalyzed by K48R (Ub mutant with lysine 48 mutated to arginine; Fig 5L, lane 1) and was further upregulated by NS1 (Fig 5L, lane 2). Additionally, MMP9 protein expressions were increased in the presense of NS1 and K63-containing Ubs, which demonstrated that NS1 specifically facilitated K63-linked polyubiquitination of MMP9 and its accumulation.

## Discussion

Understanding the mechanisms of ZIKV entry into the testes is critical to ZIKV prevention and treatment. In our study, there were three key observations: (1) MMP9 was upregulated by ZIKV infection both *in vivo* and *in vitro*; (2) MMP9 played an important role in promoting ZIKV entry into the testes by disrupting the BTB; and (3) ZIKV NS1 interacted with MMP9 and facilitated K63-linked polyubiquitination of MMP9.

Sperm production is absolutely dependent on a testicular structure called the BTB. Regulation of the BTB is particularly important and complicated. Over the past few decades, multiple factors, including steroids, nonreceptor protein kinases, and cytokines, have been found to downregulate TEER and TJP distributions in SCs *in vitro* [29]. MMP9 is also a crucial mediator of BTB restructuring [40].

MMP9 can be upregulated by multiple viral infections, triggering a series of downstream effects. For example, MMP9 induced by respiratory syncytial virus (RSV) infection enhances the multiplication and spread of RSV [41]. MMP9 also facilitates hepatitis B virus replication by repressing the interferon (IFN)-dependent signaling pathway [42]. In addition, infection by various flaviviruses can also elevate MMP9 expression. West Nile virus, which has high tropism to the brain, can induce MMP9 expression to facilitate viral entry into the brain [34]. MMP9 is also associated with endothelial glycocalyx degradation caused by dengue virus NS1 [43].

Based on the findings of the current study, we hypothesized that MMP9 may play a critical role in disrupting the BTB to facilitate ZIKV entry into the seminiferous tubules. First, we observed significant upregulation of MMP9 both *in vivo* and *in vitro* after ZIKV infection, consistent with the results of quantitative proteomic analysis using ZIKV-infected A129 mouse testes. Significant increase of MMP9 both in the sera and testis was detected as early as 6dpi at which point strong ZIKV positive signals were found in the interstitium, and much weaker but still detectable signals were found inside the seminiferous tubules, indicating a small amount of viruses had already crossed the barrier. We hypothesized the serum MMP9 circulating in the body fluid might be the major source of MMP9 in the testis at the early stage of infection, leading to mild leakage of BTB. This allows for subsequent robust spread of ZIKV inside of the seminiferous tubules and infection of its target cells in seminiferous tubules such as Sertoli cells, referred to a reservoir for ZIKV [25], which in turn produce MMP9 accelerating the disruption of BTB and ZIKV invasion of the testis. However, Siemann et al. showed that MMP9 was not upregulated in human SCs after ZIKV infection [25]. Furthermore, we explored the correlations between MMP9 and BTB permeability *in vivo* and *in vitro*. The results showed that viral loads in the testes of MMP9^-/-^ mice were lower than those in WT mice at 10dpi, even though peripheral viremia was similar in both groups of mice. Histopathology and immunofluorescence analysis revealed that MMP9^-/-^ mice had milder pathological manifestations and leukocyte infiltration and slightly less TJPs changes compared with ZIKV-infected WT mice. Additionally, in the absence of MMP9, Evans blue leakage into the testes was markedly reduced, and type Ⅳ collagen expression was largely maintained. We also used primary mSCs as an *in vitro* model to investigate the effects of ZIKV on SCB permeability. The results suggested that mSCs were indeed susceptible to ZIKV infection, consistent with the conclusions of Sheng et al. [19]. More importantly, we showed that ZIKV infection directly caused an increase in the permeability of the SCB, which was reflected by the reduced resistance values. However, when an MMP9-specific inhibitor was added, the decrease in resistance was significantly attenuated. Thus, we concluded that the hyperpermeability of the BTB could be attributed to ZIKV-induced upregulation of MMP9. In contrast, Siemann et al. used human SCs and demonstrated that ZIKV could cross the *in vitro* SCB without altering barrier permeability or the expression of TJPs [25]. The discrepancies between these two studies maybe related to the use of different cell types and viral strains. Additionally, these different results may explain why ZIKV causes varying degrees of disease in humans and mice.

Under normal conditions, mammalian testes are in an immunoprivileged environment. However, ZIKV infection can change this microenvironment into an environment with a strong antiviral response, thereby altering the permeability of the BTB [44, 45]. Siemann et al. suggested that inflammatory mediators secreted from ZIKV-infected macrophages compromised barrier integrity [25], which highlights the role of inflammatory mediators in regulating SCB permeability. In our study, increased CD45 staining in mouse testes after ZIKV infection also indicated the inflammatory response caused by ZIKV. Moreover, MMP9 can be produced and secreted by various infected cells, such as macrophages [46] and T cells [47]. Thus, MMP9 may be a crucial component in the extracellular environment of ZIKV-infected macrophages, resulting in disruption of the BTB. We believe that disruption of the BTB may be the result of the effects of both MMP9 and inflammatory cytokines. However, further studies are needed to completely elucidate the detailed mechanisms through which inflammatory mediators disrupt BTB permeability.

Currently, research on the regulation of MMP9 is still very limited [33]. In our study, we found that MMP9 mRNA and protein expression was upregulated by ZIKV infection. Retinoic acid-inducible gene I, Toll-like receptor 3, and MDA5 are the primary pattern recognition receptors that recognize ZIKV RNA in the cytoplasm [45], leading to activation of nuclear factor (NF)-κB. Moreover, the MMP9 promoter possesses one NF-κB and two activator protein-1 binding sites [33]. Therefore, we hypothesized that ZIKV may stimulate the transcriptional expression of MMP9 by activating the NF-κB binding site of MMP9. However, with regards to the complexity of upregulation of MMP9 expressions, we believe a variety of factors are involved and therefore the mechanisms merit further investigations. Recent studies have also shown that the nonstructural proteins of ZIKV are important for entry, replication, assembly, and pathogenesis. In this study, we showed that NS1, not the other proteins, upregulated MMP9 and that this effect was not observed at the transcript level. Additionally, we observed a decrease in resistance values by transfecting NS1 expression plasmids into primary mSCs, demonstrating that NS1 upregulated MMP9, resulting in increased SCB permeability.

In addition, considering that DENV NS1 has been reported to disrupt endothelial permeability [48], we also examined whether the SCB permeability would be disrupted by the recombinant ZIKV NS1 protein (S3B Fig). Interestingly, we found that NS1 can cause SCB hyperpermeability, and this effect can be eliminated by using NS1 neutralizing antibody. It was reported that ZIKV NS1 disrupts glycosaminoglycans and causes permeability in developing human placentas [49]. Thus, we speculated that the effect of NS1 protein on SCB permeability may be related to its damaging effect on glycosaminoglycans. However, further studies are needed to test this hypothesis. Moreover, we found that NS1 can also interact with MMP9 in the cell-culture supernatant, indicating that the secreted proteins might work together through interactions. However, it would be more indicative of the role of extracellular NS1 in the virus invasion of the testes by *in vivo* treatment with anti-NS1 antibodies, and it would be more evident of the function of MMP9 if we add recombinant MMP9 into MMP9 KO mice. These issues are definitely worth further investigation.

In this study, we found the intracellular overexpression of NS1 increased the endogenous MMP9 protein products with no effect on its mRNA levels, indicating a posttranslational modification might be involved. We also showed that NS1 interacted with MMP9 and that these two proteins colocalized in the cytoplasm, and NS1 promoted MMP9 stability by preventing proteasome degradation of MMP9. Ubiquitination is a post-translational modification that signals multiple processes, and K48- and K63-linked chains, the two most abundant chain types, regulate proteasomal degradation and proteasome-independent pathways, respectively [39]. Notably, we found that NS1 facilitated the K63-linked polyubiquitination of MMP9 to enhance the stability of MMP9.

**Fig 6:**
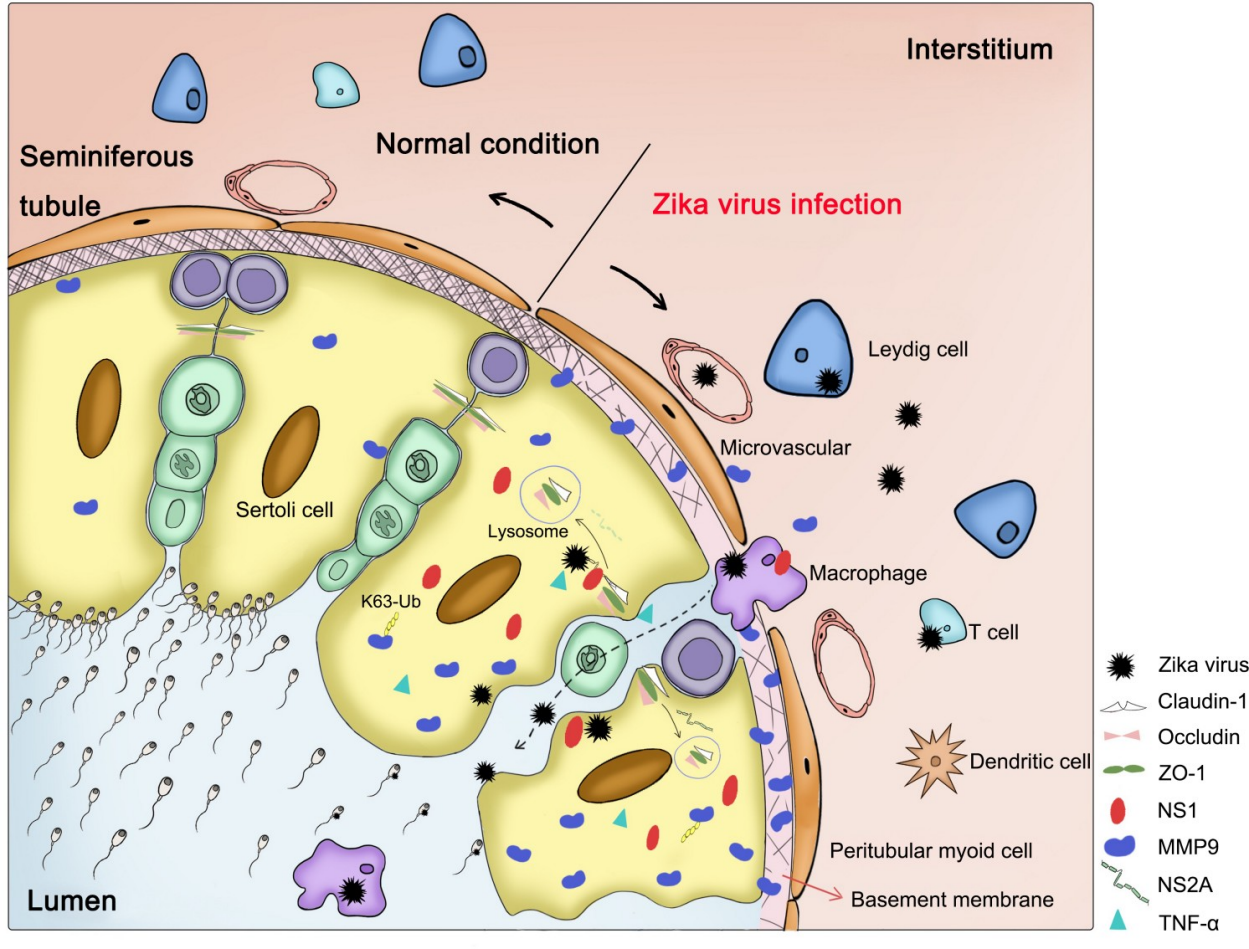
The possible mechanisms through which ZIKV enters the testes. Under normal conditions, earlier germ cells are continually separated from the blood supply into the lumen of the seminiferous tubules. The BTB, which localizes basally between adjacent SCs, is closest to the basement membrane, i.e., a type of pericellular matrix that is stabilized by a collagen type Ⅳ network. The testes are in an immunoprivileged environment. During ZIKV infection, Leydig cells, SCs, and immune cells (e.g., macrophages, T cells, and dendritic cells) are infected with ZIKV, changing the microenvironment from an immunosuppressive environment to a strong antiviral environment. Activated macrophages secrete cytokines, such as tumor necrosis factor α, which can also disrupt the BTB by degradation of TJPs, allowing ZIKV to reach and infect germ cells. During the infection course, large amounts of MMP9 in the sera or testes are induced and translocate to the basement membrane, where it degrades its target proteins, including TJPs and type Ⅳ collagen, thereby destroying the integrity of the BTB. NS2A may also interact directly with adherent junction components, targeting them for lysosomal degradation as it does in mouse radial glial cells and human brain organoids. In addition, ZIKV may be carried in the infected macrophages through the BTB into the lumen of the seminiferous tubules. Intracellular NS1 can enhance the stability of MMP9 by promoting K63-linked polyubiquitination of MMP9.

TJPs play indispensable roles in maintaining the integrity of the BTB. In our study, we found that TJPs and collagen Ⅳ were significantly decreased in ZIKV-infected WT mice, but were expressed at normal levels in MMP9^-/-^ mice, suggesting that TJPs may act as hydrolytic substrates of MMP9. Additionally, several studies have shown that ZIKV NS2A can directly interact with adherent junction components, such as ZO-1 and β-catenin, and target them for lysosomal degradation in mouse radial glial cells and human brain organoids. This phenomenon may also occur in ZIKV-infected testes; however, further studies are needed to test this hypothesis [50]. In addition, activated and infected macrophages can also interact with SCs and infiltrate the lumen of seminiferous tubules [44]. TNF-α, a pro-inflammatory cytokine, has also been shown to negatively affect SCB/ BTB function by upregulating proteases [51]. We have summarized the possible mechanisms through which ZIKV gained entry into the testes in Fig 6.

In conclusion, our findings improved our understanding of the molecular mechanisms underlying testis invasion by ZIKV. The genital tropism of ZIKV and persistence of infectious ZIKV in seminal fluids, as free particles or associated with spermatozoa, leukocytes, epithelial cells or other cell types within semen increase the risk of sexual transmission and offspring malformation, making this disease a major public health concern. Thus, the results of our study also provide insights into potential strategies for preventing ZIKV infection in male genital organs and attenuating its pathological effects during infection.

## Materials and methods

### Ethics statement

All animal handling procedures were performed in compliance with Chinese Animal Protection Act and the National Research Council criteria. The experiments and protocols were approved by the Committee on the Ethics of Animal Experiments of the Institute of Microbiology, Chinese Academy of Sciences (IMCAS) under permit SQIMCAS2018039. All animal experiments were conducted under isoflurane anesthesia to minimize animal suffering. Studies with ZIKV were conducted under biosafety level 2 and Animal Biosafety Level 3 (BSL3) containment.

### Cells and virus

HeLa cells (American Type Culture Collection [ATCC], Manassas, VA, USA; #CCL-2), adenocarcinomic human alveolar basal epithelial cells (A549, ATCC; #CCL-185), and human embryonic kidney cells (HEK293T) were cultured in Dulbecco’s modified Eagle’s medium (DMEM; Gibco, Grand Island, NY, USA) supplemented with 10% fetal bovine serum (FBS), penicillin (100 μg/mL), and streptomycin sulfate (100 μg/mL) at 37°C in an atmosphere containing 5% CO_2_. Primary mouse Sertoli cells (mSCs) were isolated from C57BL/6 mice and primary mouse embryonic fibroblasts (MEFs) were isolated from IFNα/β receptor-deficient mice (A129). ZIKV (ZIKA-SMGC-1; GenBank accession number: KX266255) was kindly provided by Dr. George F. Gao (Institute of Microbiology, Chinese Academy of Sciences, Beijing, China). ZIKV was propagated in *Aedes albopictus* mosquito cells (C6/36; ATCC; #CRL-1660) as described previously [1].

### Reagents and antibodies

Trypsin, DMEM, and DMEM/F12 were obtained from Gibco. Lipofectamine 3000 and normal mouse immunoglobulin G (IgG) were purchased from Invitrogen Corporation (Carlsbad, CA, USA). Rabbit anti-ZIKV NS1 (1:1000; catalog no. GTX133307) and COL4A1 antibodies (1:1000; catalog no. GTX130215) were purchased from GeneTex (USA). Anti-Flag (F3165) antibody (1:2000), anti-HA (H9658) antibody, and monoclonal mouse anti-glyceraldehyde 3-phosphate dehydrogenase antibody (GAPDH; 1:5000; G9295) were purchased from Sigma-Aldrich Corp. (St. Louis, MO, USA). Anti-ZO-1 antibody (catalog no. sc-33725) was purchased from Santa Cruz Biotechnology (Dallas, TX, USA). Anti-ZIKV antibody (FITC-Z6) and ZIKV-specific neutralizing antibody-Z23 (targeting the E protein) were kindly provided by Dr. George F. Gao (Institute of Microbiology, Chinese Academy of Sciences, Beijing, China). Anti-green fluorescent protein (GFP) antibody (catalog no. 50430-2-Ap), anti-occludin antibody (catalog no. 27260-1-AP), and anti-claudin-1 antibody (catalog no. 13050-1-AP) were purchased from Proteintech Group (Chicago, IL, USA). Anti-human MMP9 antibody (catalog no.13667) was purchased from Cell Signaling Technology (Boston, MA, USA), anti-mouse MMP9 antibody (catalog no. AF909) and recombinant mouse MMP9 proteins (catalog no. 909-MM) were purchased from R&D Systems (Minneapolis, MN, USA). Ifnar-blocking mouse monoclonal antibodies (MAR1-5A3, BE0241) and anti-mouse IgG1 isotype control (MOPC-21, BE0083) were purchased from Bio X Cell. CCK8 Kit was purchased from biosharp (BS350B). NS1 protein and anti-NS1 polyclonal antibodies (neutralization of NS1) were kindly provided by Dr. Gong Cheng (Tsinghua-Peking Center for Life Sciences, School of Medicine, Tsinghua University, Beijing, China).

### Mouse experiments

A129 mice (IFNα/β receptor-deficient) were purchased from the Institute of Laboratory Animal Science, Chinese Academy of Medical Sciences and Peking Union Medical College. C57BL/6 mice were purchased from the Hubei Provincial Center for Disease Control and Prevention (Wuhan, China). C57BL/6 MMP9^-/-^ mice were kindly provided by Dr. Meng-cheng Luo.

For ZIKV infection, 8 week-old male A129 mice were infected with 1 × 10^6^ PFU of ZIKV by intraperitoneal injection (n = 5 for each group). Mice administered phosphate-buffered saline (PBS) served as a control. At the indicated times postinfection, the mice were euthanized, and the whole blood and testes were harvested for qRT-PCR, immunofluorescence analyses, gelatin zymography assays, and western blotting.

WT or MMP9^-/-^ C57BL/6 mice (6–7 weeks of age) were treated with Ifnar-blocking mouse monoclonal antibodies by intraperitoneal injection four times: 2 mg at 1 day prior ZIKV infection (day -1), 0.5 mg at 1 day after infection (day 1), 0.5 mg at 3 days after infection (day 3), and 0.5 mg at 5 days after infection (day 5). Mice were intraperitoneally injected with 1 × 10^7^ PFU of ZIKV on day 0 and were euthanized on day 10. WT C57BL/6 mice (6–7 weeks of age) treated with anti-mouse IgG1 isotype control antibodies use the same antibody dose and virus dose as above. The whole blood and testes were harvested for analysis. Viral loads in the whole blood and testes were determined by qRT-PCR and western blotting. Testis tissues were sectioned, fixed with 4% paraformaldehyde (PFA), and stained with H&E or specific antibodies.

### qRT-PCR

Total RNA from cells was extracted with TRIzol reagent (Invitrogen), and mRNA was then subjected to reverse transcription using Moloney murine leukemia virus reverse transcriptase (Promega, Madison, WI, USA). qRT-PCR was performed with iTaq Universal SYBR Green Supermix (Bio-Rad Laboratories, Inc., Hercules, CA, USA) on a CFX Connect Real-Time System. A probe-based assay was performed to quantify viral RNA copy number by TaqMan qPCR amplification of ZIKV E gene. A standard curve was generated using serially diluted expression plasmids containing the coding sequence of ZIKV E. Primers used to amplify corresponding genes were as follows: human MMP9, 5′-CCTCTGGAGGTTCGACGTG-3′ (forward) and 5′-AACTCACGCGCCAGTAGAAG-3′ (reverse); mouse Mmp9, 5′-TGGACGCGACCGTAGTTG-3′ (forward) and 5′-GCTTGCCCAGGAAGACGAA-3′ (reverse); mouse Mmp2, 5′-GAGACCGCTATGTCCACTGT-3′ (forward) and 5′-CTTGTTGCCCAGGAAAGTGAAG-3′ (reverse); ZIKV, 5′-TGAYAAGCARTCAGACAC-3′ (forward), 5′-TCACCARRCTCCCTTTGC-3′ (reverse) and probe 5'-FAM-GTGGAYAGAGGYTGGGGAAA-TAMRA-3'; human GAPDH, 5′-ATGACATCAAGAAGGTGGTG-3′ (forward) and 5′-CATACCAGGAAATGAGCTTG-3′ (reverse); mouse Gapdh, 5′-TGTGTCCGTCGTGGATCTGA-3′ (forward) and 5′-TTGCTGTTGAAGTCGCAGGAG-3′ (reverse).

### H&E analyses and immunofluorescence staining

ZIKV-challenged mice were euthanized at 2, 4, 6, 8, and 10 days postinfection, and testes were fixed in 4% PFA. Testes were either cryosectioned or sectioned in paraffin. For H&E analyses, paraffin sections (5μm) were stained with H&E. Normal mice served as controls. For immunofluorescence staining, frozen testes sections (6μm) were stained with specific antibodies and then with fluorophore-labeled secondary antibodies. Sections were then imaged using a Zeiss LSM510 laser scanning confocal microscope. Images were processed using Adobe Photoshop (Adobe, CA, USA).

### Western blotting and Co-IP assay

Briefly, after treatment, freshly isolated testes and whole-cell lysates were homogenized and lysed for 45 min in lysis buffer (50 mM Tris-HCl [pH7.5], 300 mM NaCl, 1% Triton-X, 5mM ethylenediaminetetraacetic acid, 10% glycerol [v/v], and 1× protease inhibitor [Roche]) on ice. Lysates were immunoprecipitated with control goat IgG (ProteinTech), anti-Flag antibodies (Sigma; cat. no. F3165), or anti-MMP9 antibodies (R&D Systems; cat. no. AF909) with Protein-G Sepharose (GE Healthcare, Milwaukee, WI, USA). Protein concentrations were estimated using a BCA Protein Assay Kit (Thermo Fisher Scientific, USA). Proteins were separated using sodium dodecyl sulfate polyacrylamide gel electrophoresis (SDS-PAGE) on 8–10% gels and then transferred to polyvinylidene difluoride membranes (Millipore Corp). The membranes were blocked with 5% skim milk in PBS with 0.1% Tween 20 and incubated with antibodies. Protein bands were detected using a QuickGel 6100 Imager (Monad).

### Enzyme-linked immunosorbent assay (ELISA) and cell viability assay

The concentrations of MMP9 in the serum were measured using mouse MMP9 ELISA Kits (BlueGene Biotech, Shanghai, China) following the manufacturer’s instructions. And the viability of the primary mSCs were measured using CCK8 Kits following the manufacturer’s instructions.

### Gelatin zymography

Gelatin zymography assays were performed as previously described [34]. Briefly, samples were resolved on SDS-PAGE (7.5% [w/v] polyacrylamide) gels containing 0.1% (w/v) gelatin (Sigma) in running buffer (25 mM Tris, 250 mM glycine, 1% SDS) at room temperature. The gels were washed with 50 mM Tris-HCl (pH 7.5) containing 2.5% Triton X-100 three times (30 min each) and then incubated in reaction buffer (50 mM Tris-HCl [pH 7.5] containing 10 mM CaCl_2_, 1 μM ZnCl_2_, 0.02% [w/v] BrijH-35, 0.01% [w/v] NaN_3_] at 37°C for 24–48 h. Protein bands were visualized by 0.25% (w/v) Coomassie brilliant blue R-250 (Sigma) staining followed by destaining. Gelatinase activity was detected as unstained bands on a blue background.

### Evans blue BTB permeability assay

WT C57BL/6 mice were treated with Ifnar-blocking mouse monoclonal antibody and infected with ZIKV, as previously described [1]. Ten days postinfection, mice were injected intravenously with 0.5% Evans blue solution (200 μL/mouse; Vetec, Rio de Janeiro, Brazil). After 1 h, mice were perfused with PBS until the drainage was colorless. The testes were removed, weighed, placed in 1 mL formamide (Vetec), and incubated at 60°C for 24–36h for stain extraction. Subsequently, samples were centrifuged at 13000 × *g* for 10 min, the supernatants (formamide extracts) were removed carefully, and the absorbance at 611 nm was measured using a spectrometer. Formamide extracts from clean mouse testes were used as a blank. The blue coloration of testes was a qualitative indicator of BTB permeability.

### Isolation of SCs

As described previously [38], SCs were isolated from the testes of 6–10-day-old male WT C57BL/6 mice. Briefly, freshly isolated testes were washed with precooled PBS, the white membrane outside the testes was gently removed, and the testes were then into small pieces. Trypsin (0.125%) was added for digestion at 37°C for 2 min, and the digestion was then terminated with DMEM/F12 containing FBS. The cells were washed three times with DMEM/F12, with centrifugation at 900 rpm for 5 min each wash. Subsequently, the medium was removed, and the cells were resuspended in fresh 10% FBS-containing DMEM/F12 and seeded into cell culture flasks. After 48h, the cells were treated with 20 mM Tris (pH7.4) for 20 min to remove residual germ cells.

### TEER

As described previously [25, 38], to evaluate the effects of MMP9 on the integrity of the SCB, 2.5 × 10^5^ primary mSCs were plated on a 12-well Transwell polycarbonate membrane system (Corning Life Sciences, Corning, NY, USA) with a 0.4-μm pore size and a 1.12-cm^2^ membrane surface. The integrity of this *in vitro* SCB model was determined by measuring TEER using an epithelial volt/ohm meter (Evomx) with “chopstick” electrodes (World Precision Instrument) every day from day 2 after seeding. Experiments were performed with a high resistance until TEER values crossed 50 Ω • cm^2^, depending on the cell type, indicating 100% cell confluency [38]. Subsequently, cells were infected with ZIKV (MOI = 5) and then treated with or without the specific MMP9 inhibitor JNJ0966 for 1h. The inserts were gently washed three times with medium to remove unbound virus, and cells were treated again with the inhibitor. The TEER was measured 12, 24, 36, 48, and 72h after infection. In addition, the activated MMP9 protein with or without JNJ0966 was added to the upper chambers, and resistance values were measured at the indicated times. The SCB permeability was expressed as the relative TEER, which represents the ratio of resistance values (Ω) as follows: (Ω experimental condition –Ω medium alone) / (Ω untreated SCs –Ω medium alone).

### Confocal microscopy

HEK293T and HeLa cells were transfected with the indicated plasmids (500 ng) for 24h and then fixed in 4% PFA at room temperature for 15 min. After washing three times with PBS, the cells were permeabilized with wash buffer (PBS containing 0.1% Triton X-100) for 5 min, washed three times with PBS, and finally blocked with PBS containing 5% bovine serum albumin for 1 h. The cells were then incubated with the primary antibody overnight at 4°C, incubated with FITC-conjugated donkey anti-mouse IgG for 1 h, washed with wash buffer three times, incubated with 4′,6-diamidino-2-phenylindole (DAPI) solution for 5 min, and then washed three more times with PBS. Finally, the cells were analyzed using a confocal laser scanning microscope (Fluo View FV1000; Olympus, Tokyo, Japan).

### Statistical analyses

All analyses were performed using GraphPad Prism statistical software. All numerical data are presented as the means ± standard errors of the means (SEMs). Results with P values of less than 0.05 were considered significant. All experiments were repeated at least three times. Data were analyzed by one-way analysis of variance (ANOVA), two-tailed Student’s t-test, or two-way ANOVA as indicated for different experiments.

## Supporting information

S1 Fig **(A)** Protein level of MMP9 in the testes of the MMP9^-/-^ mice were quantified by western blotting. The testis from C57BL/6 WT mouse was used as a positive control. **(B)** C57BL/6 WT and MMP9^-/-^ male mice (6–7 weeks old) treated with Ifnar-blocking mouse monoclonal antibodies were infected intraperitoneally with ZIKV (1 × 10^7^ PFU). The C57BL/6 WT mice treated with isotype control antibodies were also infected intraperitoneally with ZIKV (1 × 10^7^ PFU) as a mock control. Protein levels of occludin, ZO-1, collagen Ⅳ, MMP9, NS1, and GAPDH in the testes were quantified by western blotting with the indicated antibodies at 10 dpi.
(TIF)

S2 Fig **(A)** HEK293T cells were infected with ZIKV at different MOI for 30h. Uninfected cells were used as a mock control. MMP9 mRNA levels were measured by quantitative RT-PCR, MMP9 protein levels were examined by western blotting. Data are expressed as means ± SEMs of three separate experiments. *P< 0.05; **P< 0.01; ***P< 0.001, ****P< 0.0001. ns, not significant (one-way ANOVA). **(B)** The primary mSCs were non-treated, infected with ZIKV (MOI = 5), treated with JNJ0966 (1uM) or treated with ZIKV (MOI = 5) and JNJ0966 (1uM) together. MMP9 proteinase activity in the supernatants was determined by gelatin zymography assays. **(C)** Cell viability of mock- and ZIKV-infected primary mSCs was assessed via CCK8 Kit at different time points after ZIKV infection (MOI of 5). **(D)** Cell viability of mock- and activated MMP9 protein-treated primary mSCs was assessed via CCK8 Kit. Data are expressed as means ± SEMs of three independent experiments. ns, not significant (one-way ANOVA).
(TIF)

S3 Fig **(A)** MMP9 secretion in the culture supernatants of cells treated with recombinant NS1 protein. Different doses of recombinant NS1 protein were added to the cell-culture supernatants of primary mSCs, and determined by western blotting after incubation for 36h. **(B)** Monolayers of primary mSCs grown on Transwell inserts were incubated for 72h with both NS1 (5μg/mL) and anti-NS1 serum (1:100 dilution), NS1 alone (5μg/mL), anti-NS1 serum alone (1:100 dilution), both NS1 (5μg/mL) and anti-E antibody (5μg/mL), anti-E antibody alone (5μg/mL) and TEER (ohm) was measured at indicated time points. TNF-α (1ng/mL) was used as positive control. Data are expressed as means ± SEMs of three independent experiments.
(TIF)

S4 Fig HEK293T cells were cotransfected with empty vector, Flag-NS1 and HA-MMP9, cell culture supernatants were immunoprecipitated with anti-HA **(A)** or anti-Flag **(B)** antibodies. The immunoprecipitates and supernatants were analyzed by western blotting with anti-Flag and anti-HA antibodies.
(TIF)
