# Supplementary figures and images for: Matrix metalloproteinase 9 facilitates Zika virus invasion of the testis by modulating the integrity of the blood-testis barrier
Source: PLoS Pathog. 2020 Apr 17;16(4):e1008509. doi: 10.1371/journal.ppat.1008509 (PMC7190178; doi:10.1371/journal.ppat.1008509)

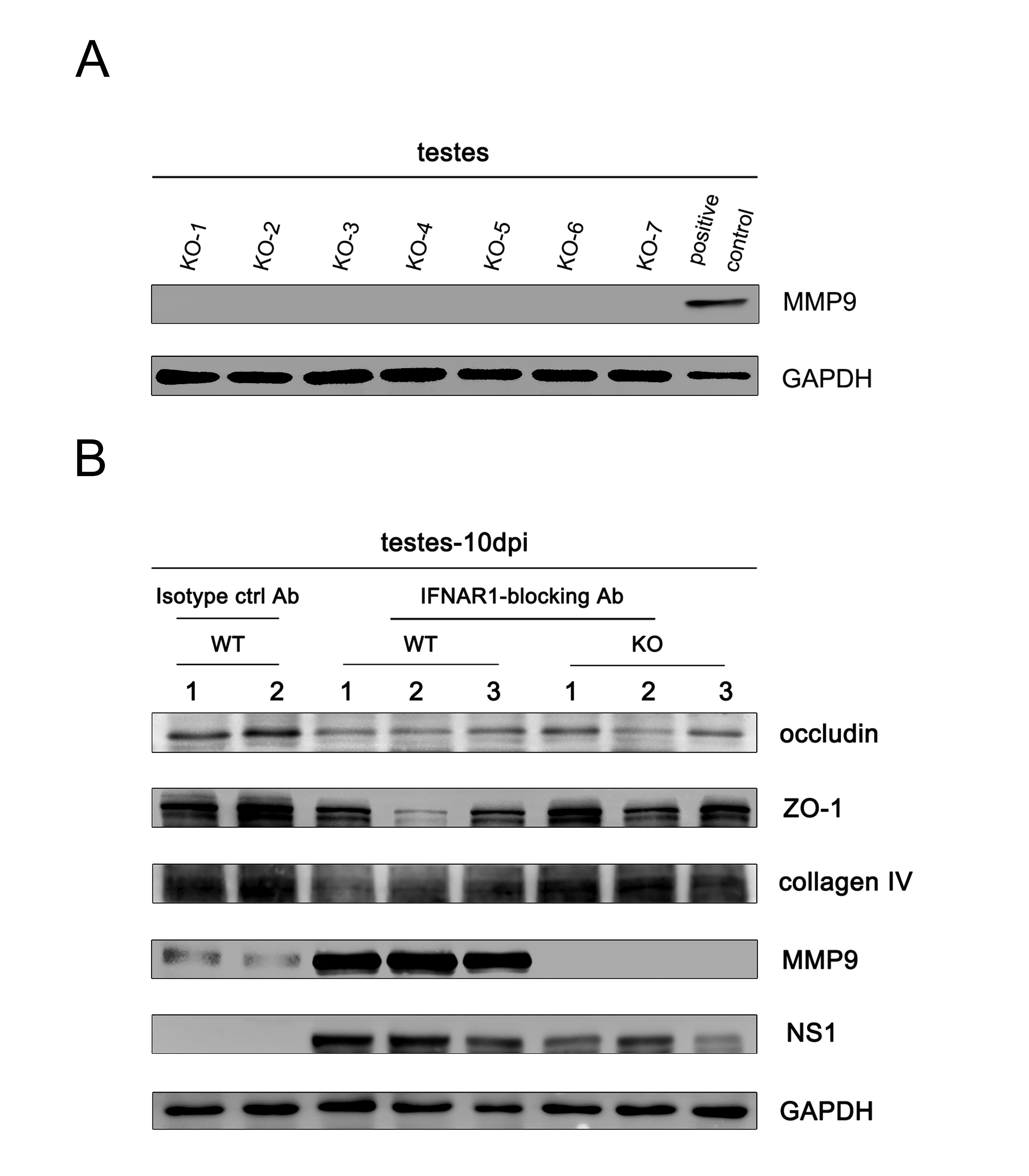

Supplement: S1 Fig — (A) Protein level of MMP9 in the testes of the MMP9-/- mice were quantified by western blotting. The testis from C57BL/6 WT mouse was used as a positive control. (B) C57BL/6 WT and MMP9-/- male mice (6–7 weeks old) treated with Ifnar-blocking mouse monoclonal antibodies were infected intraperitoneally with ZIKV (1 × 10^7^ PFU). The C57BL/6 WT mice treated with isotype control antibodies were also infected intraperitoneally with ZIKV (1 × 10^7^ PFU) as a mock control. Protein levels of occludin, ZO-1, collagen Ⅳ, MMP9, NS1, and GAPDH in the testes were quantified by western blotting with the indicated antibodies at 10 dpi. (TIF) [file ppat.1008509.s001.tif]

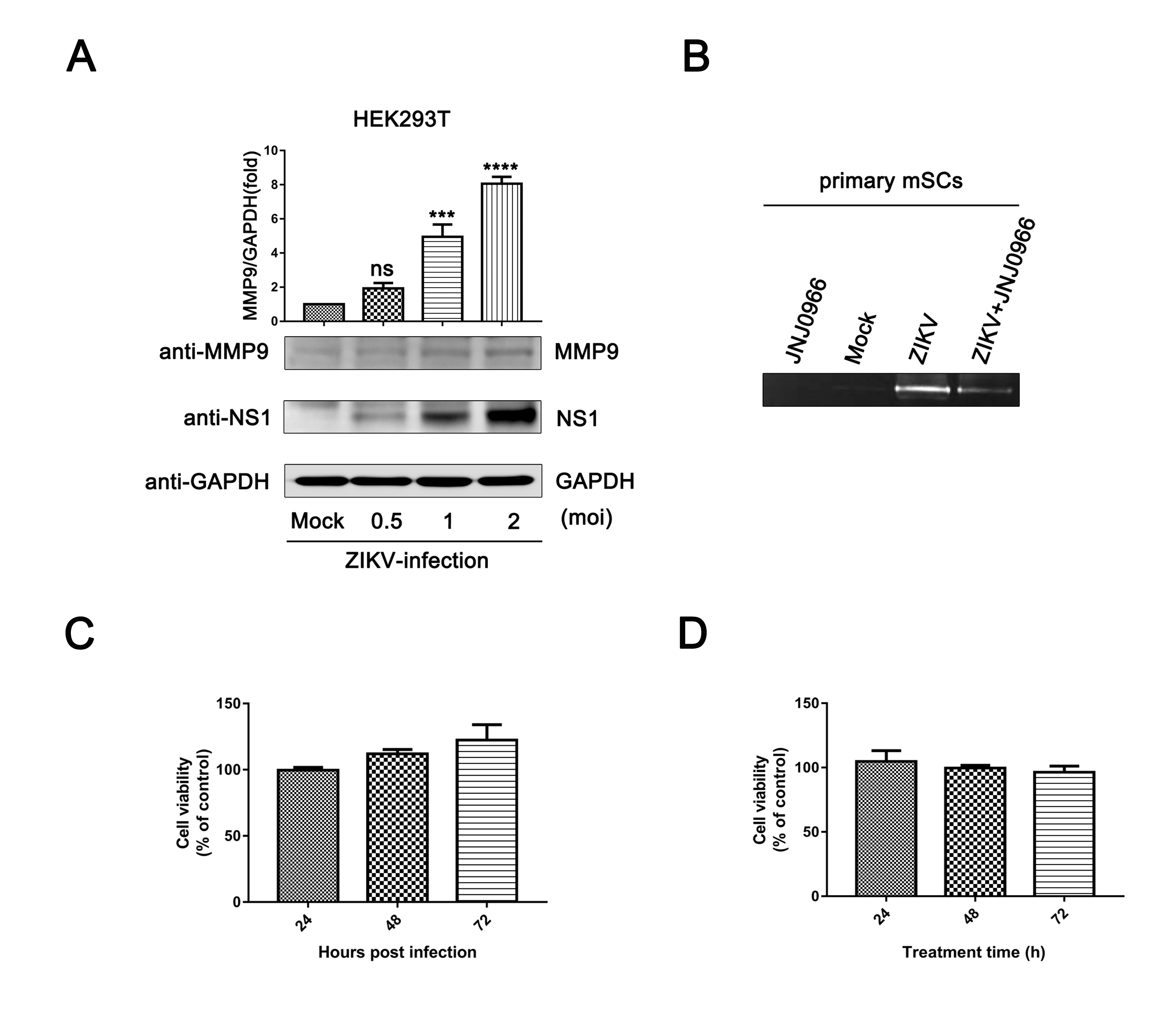

Supplement: S2 Fig — (A) HEK293T cells were infected with ZIKV at different MOI for 30h. Uninfected cells were used as a mock control. MMP9 mRNA levels were measured by quantitative RT-PCR, MMP9 protein levels were examined by western blotting. Data are expressed as means ± SEMs of three separate experiments. *P< 0.05; **P< 0.01; ***P< 0.001, ****P< 0.0001. ns, not significant (one-way ANOVA). (B) The primary mSCs were non-treated, infected with ZIKV (MOI = 5), treated with JNJ0966 (1uM) or treated with ZIKV (MOI = 5) and JNJ0966 (1uM) together. MMP9 proteinase activity in the supernatants was determined by gelatin zymography assays. (C) Cell viability of mock- and ZIKV-infected primary mSCs was assessed via CCK8 Kit at different time points after ZIKV infection (MOI of 5). (D) Cell viability of mock- and activated MMP9 protein-treated primary mSCs was assessed via CCK8 Kit. Data are expressed as means ± SEMs of three independent experiments. ns, not significant (one-way ANOVA). (TIF) [file ppat.1008509.s002.tif]

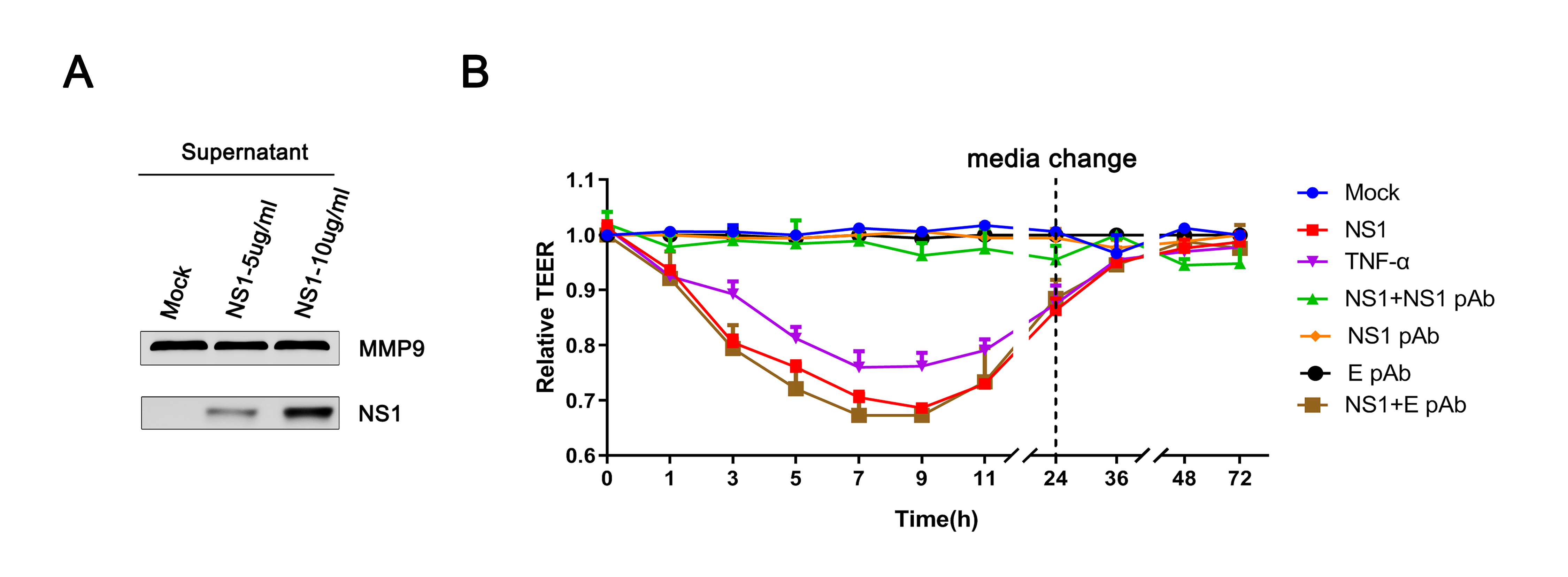

Supplement: S3 Fig — (A) MMP9 secretion in the culture supernatants of cells treated with recombinant NS1 protein. Different doses of recombinant NS1 protein were added to the cell-culture supernatants of primary mSCs, and determined by western blotting after incubation for 36h. (B) Monolayers of primary mSCs grown on Transwell inserts were incubated for 72h with both NS1 (5μg/mL) and anti-NS1 serum (1:100 dilution), NS1 alone (5μg/mL), anti-NS1 serum alone (1:100 dilution), both NS1 (5μg/mL) and anti-E antibody (5μg/mL), anti-E antibody alone (5μg/mL) and TEER (ohm) was measured at indicated time points. TNF-α (1ng/mL) was used as positive control. Data are expressed as means ± SEMs of three independent experiments. (TIF) [file ppat.1008509.s003.tif]

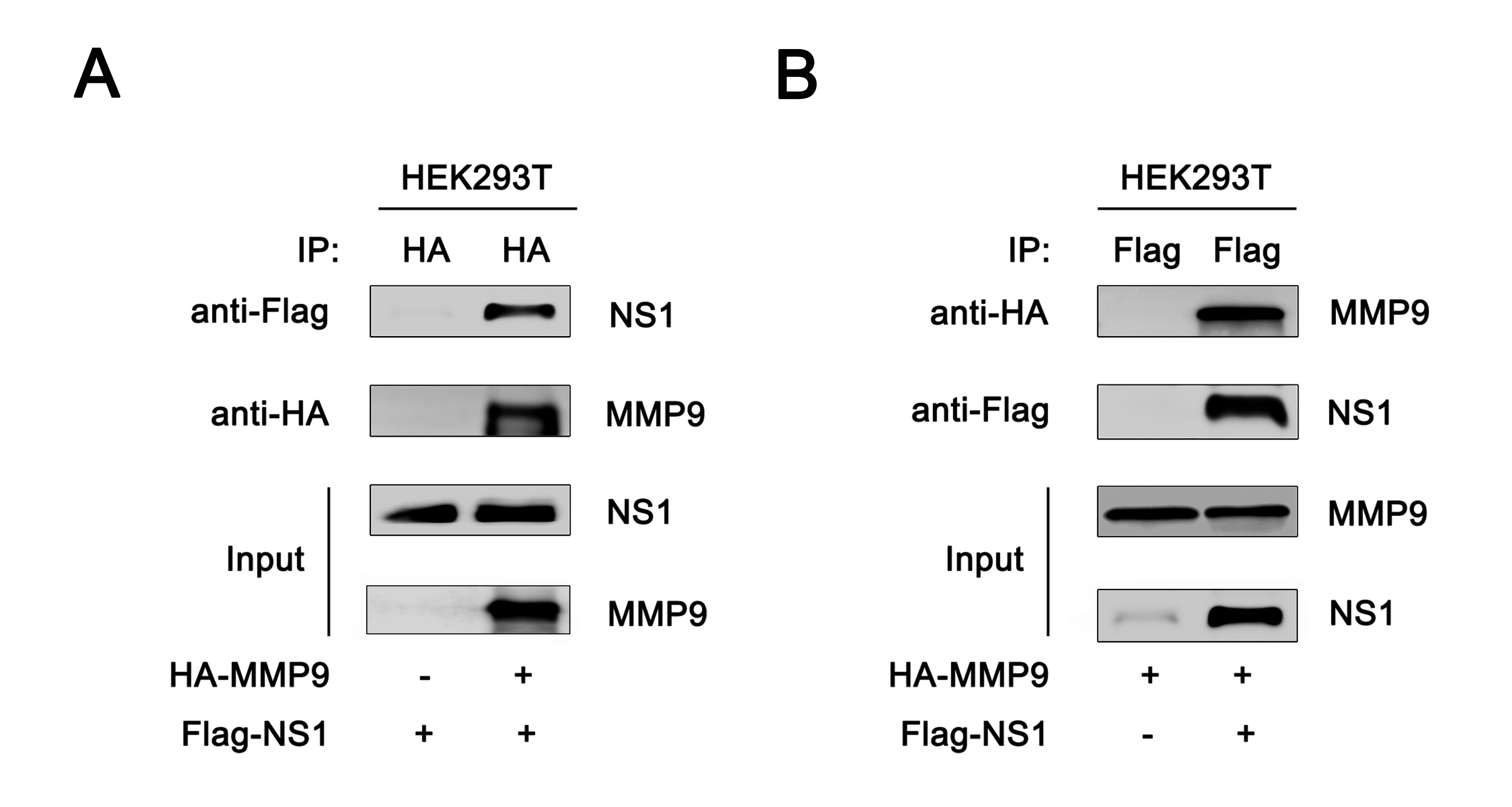

Supplement: S4 Fig — HEK293T cells were cotransfected with empty vector, Flag-NS1 and HA-MMP9, cell culture supernatants were immunoprecipitated with anti-HA (A) or anti-Flag (B) antibodies. The immunoprecipitates and supernatants were analyzed by western blotting with anti-Flag and anti-HA antibodies. (TIF) [file ppat.1008509.s004.tif]
